# Supplementary material for: Si–Fe–C–N Coatings for Biomedical Applications: A Combinatorial Approach
Source: Materials (Basel). 2020 Apr 30;13(9):2074. doi: 10.3390/ma13092074 (PMC7254256; doi:10.3390/ma13092074)
Supplement: Supplementary file 1 [file materials-13-02074-s001.pdf]

materials

MDPI

Supplementary Information

Si–Fe–C–N Coatings for Biomedical Applications: A Combinatorial Approach

Charlotte Skjöldebrand [1], Gry Hulsart-Billström [2], Håkan Engqvist [1] and Cecilia Persson [1,*]

[1] Department of Materials Science and Engineering, Faculty of Science and Technology, Uppsala University, 752 37 Uppsala, Sweden; charlotte.skjoldebrand@angstrom.uu.se (C.S.); hakan.engqvist@angstrom.uu.se (H.E.)
[2] Department of Surgical Sciences, Faculty of Medicine and Pharmacy, Uppsala University, 751 83 Uppsala, Sweden; gry.hulsart@surgsci.uu.se
* Correspondence:cecilia.persson@angstrom.uu.se

1. Significant Correlations

Graphs depicting statistically significant correlations are shown here with trend lines (Figure S1–S22).

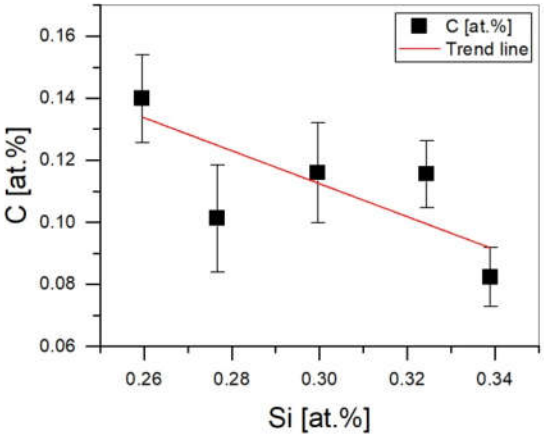

Figure S1. C as a function of Si.

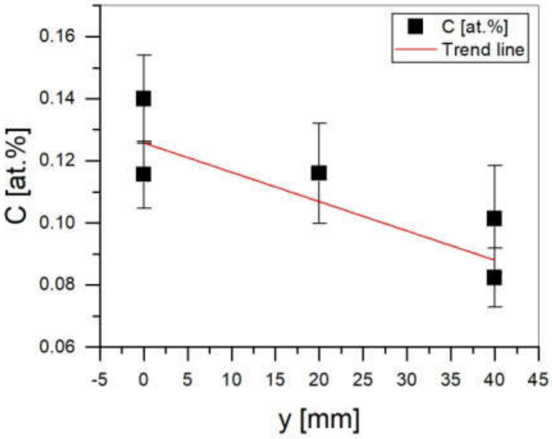

Figure S2. C as a function of y.

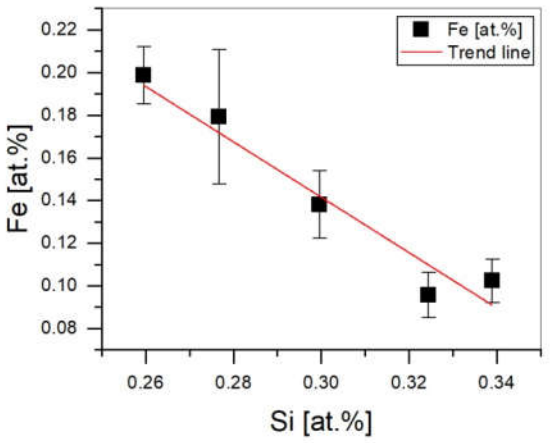

Figure S3. Fe as a function of Si.

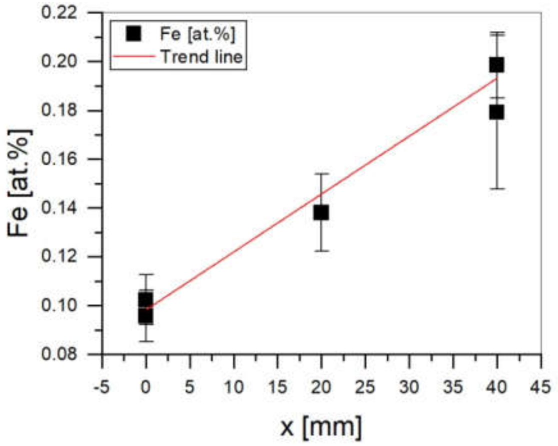

Figure S4. Fe as a function of x.

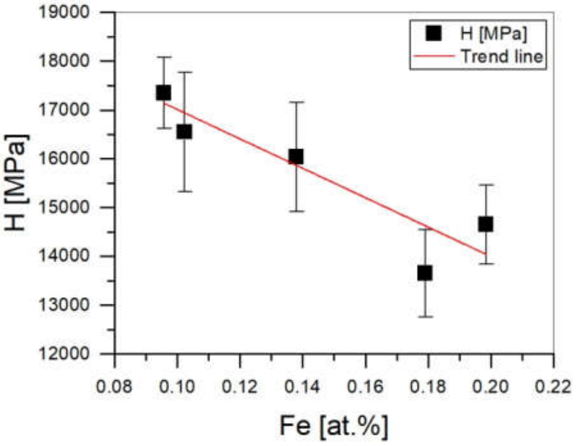

Figure S5. H as a function of Fe.

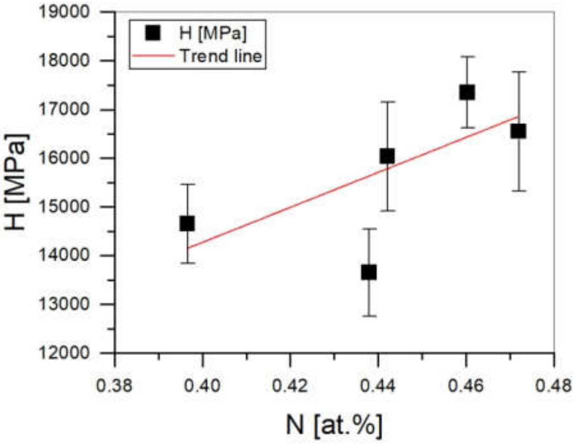

Figure S6. H as a function of N.

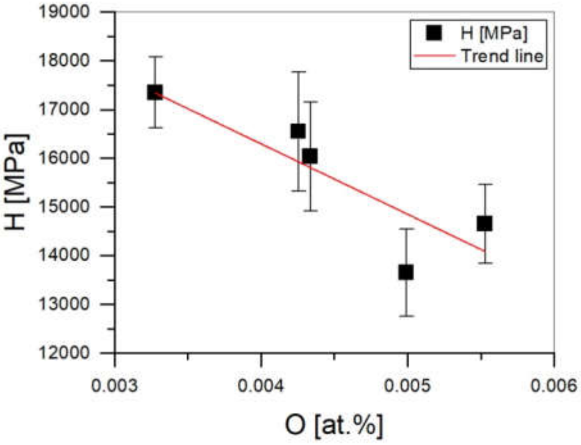

Figure S7. H as a function of O.

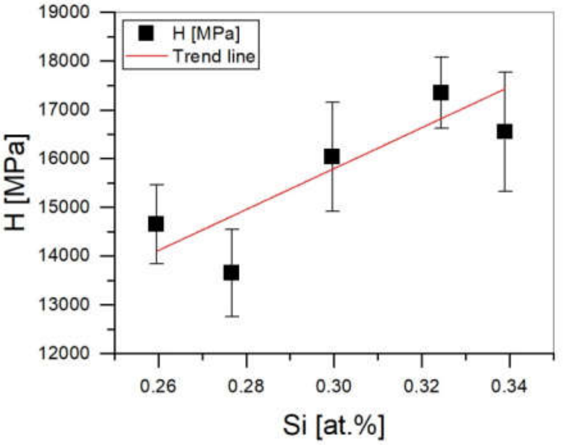

Figure S8. H as a function of Si.

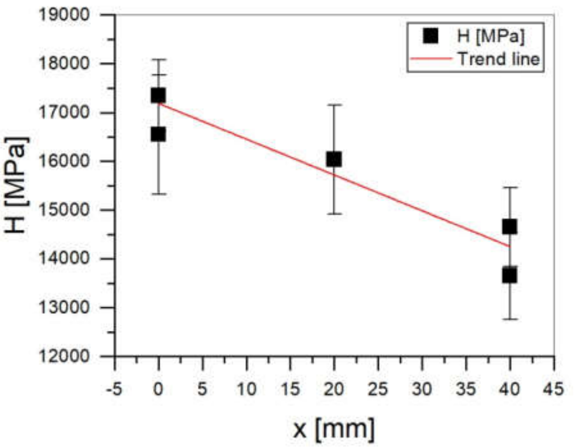

Figure S9. H as a function of x.

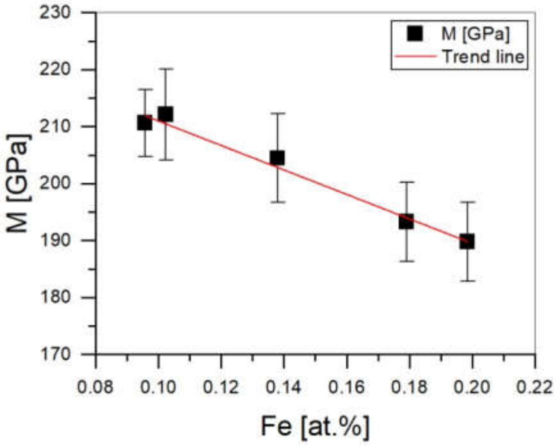

Figure S10. M as a function of Fe.

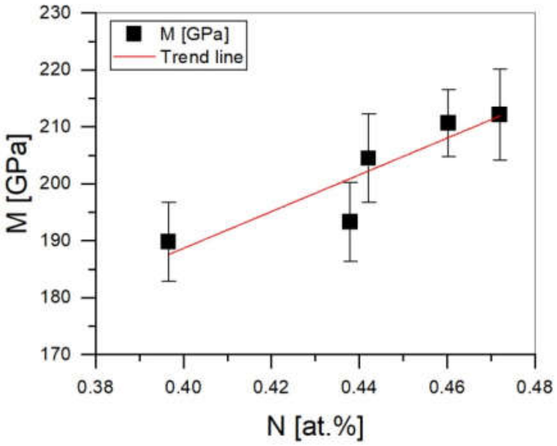

Figure S11. M as a function of N.

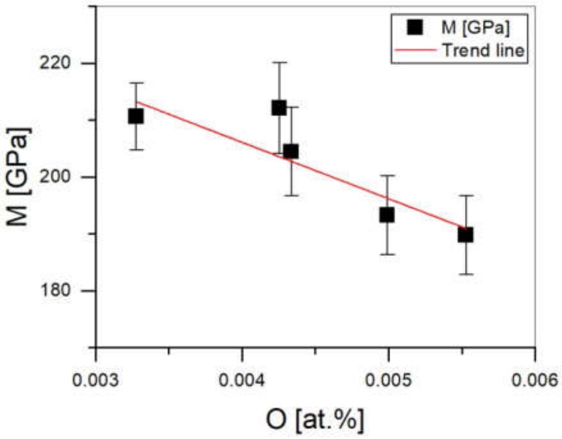

Figure S12. M as a function of O.

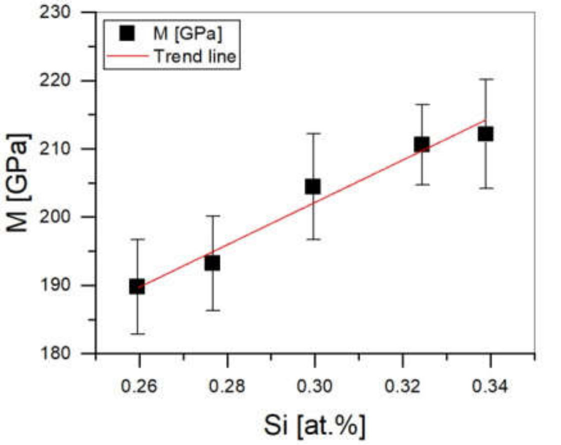

Figure S13. M as a function of Si.

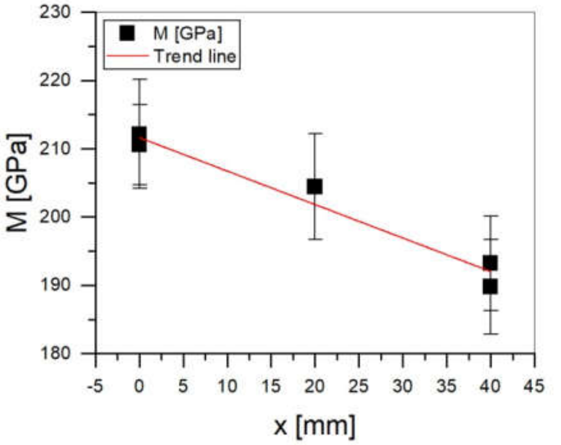

Figure S14. M as a function of x.

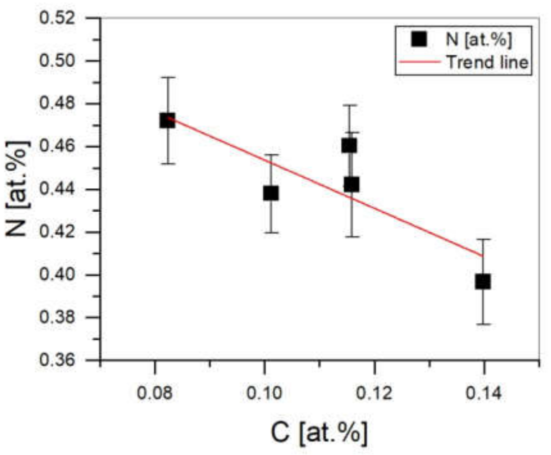

Figure S15. N as a function of C.

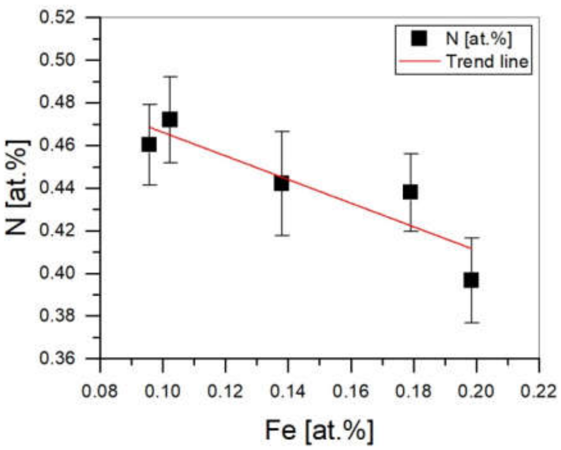

Figure S16. N as a function of Fe.

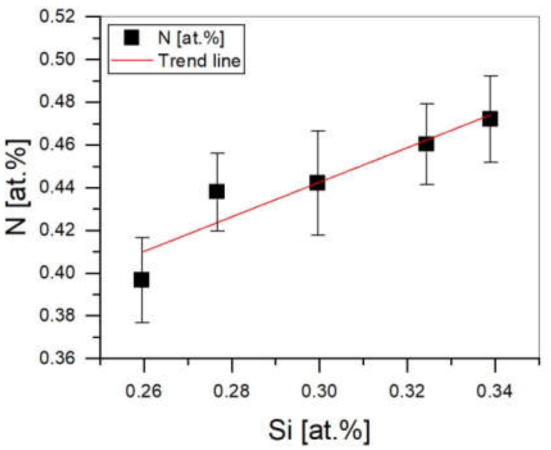

Figure S17. N as a function of Si.

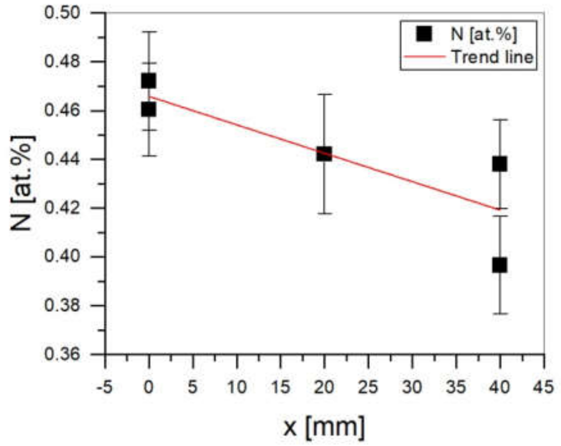

Figure S18. N as a function of x.

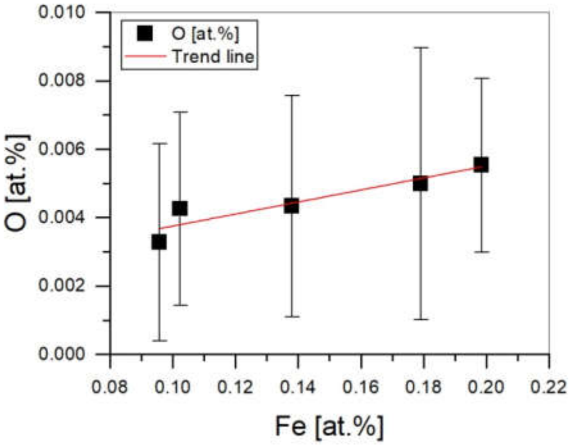

Figure S19. O as a function of Fe.

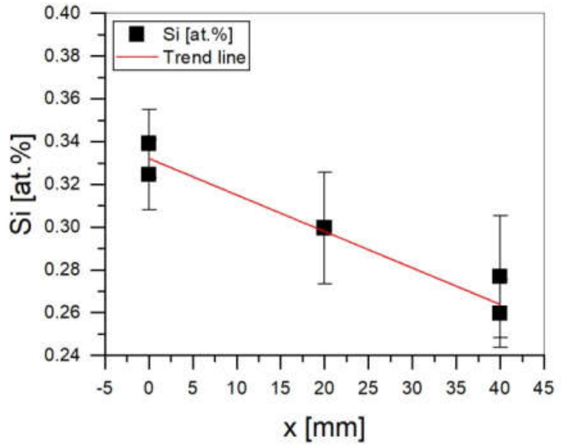

Figure S20. Si as a function of x.

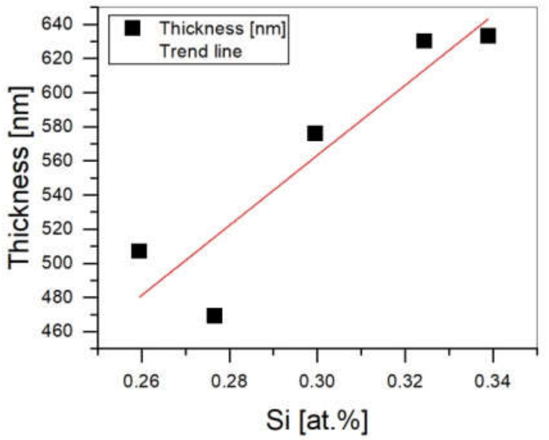

Figure S21. Thickness as a function of Si.

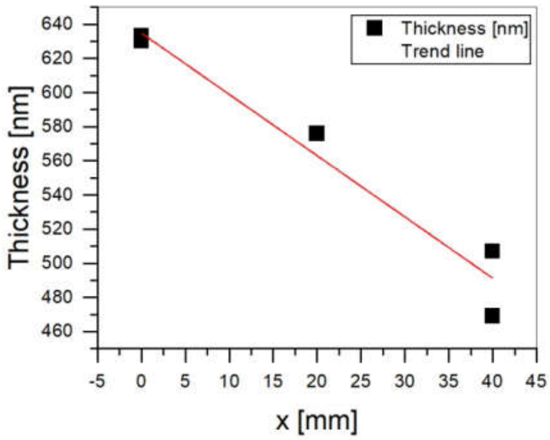

Figure S22. Thickness as a function of x.

2. Nanoindentation

Silicon is known to undergo a load induced phase transformation, which can be detected in a load displacement curved obtained using nanoindentation. It is visible as a change in the unloading of the indent. When comparing the uncoated Si wafer substrate to the coated points (**Error! Reference source not found.**) it is noticeable that the coating does not show a similar recovery and therefore indicating that the influence of the substrate is negligible.

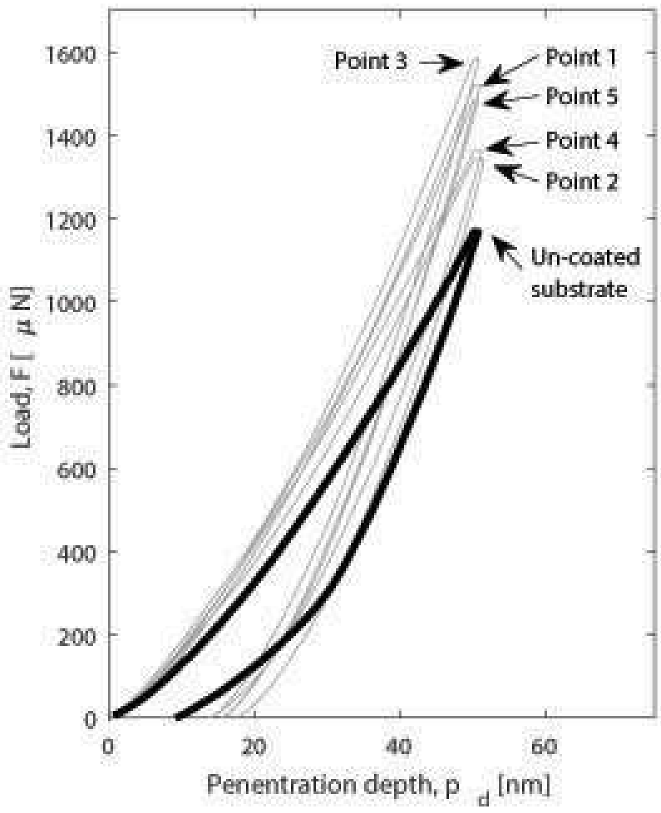

Figure S23. Load displacement curves for all investigated points (1–5) as well as an uncoated substrate.

© 2020 by the authors. Licensee MDPI, Basel, Switzerland. This article is an open access article distributed under the terms and conditions of the Creative Commons Attribution (CC BY) license (http://creativecommons.org/licenses/by/4.0/).
